# Partial oxidation of methane to methanol on boron nitride at near critical acetonitrile

**DOI:** 10.1038/s41598-022-12639-x

**Published:** 2022-05-20

**Authors:** Tharindu Kankanam Kapuge, Ehsan Moharreri, Inosh Perera, Nicholas Eddy, David Kriz, Nathaniel Nisly, Seth Shuster, Partha Nandi, Steven L. Suib

**Affiliations:** 1 Department of Chemistry, University of Connecticut, Storrs, CT 06269 USA; 2 Institute of Material Science, University of Connecticut, Storrs, CT 06269 USA; 3 Corporate Strategic Research, ExxonMobil Research and Engineering, Annandale, NJ 08801 USA

**Keywords:** Catalysis, Energy, Inorganic chemistry, Materials chemistry

## Abstract

Direct catalytic conversion of methane to methanol with O_2_ has been a fundamental challenge in unlocking abundant natural gas supplies. Metal-free methane conversion with 17% methanol yield based on the limiting reagent O_2_ at 275 °C was achieved with near supercritical acetonitrile in the presence of boron nitride. Reaction temperature, catalyst loading, dwell time, methane–oxygen molar ratio, and solvent-oxygen molar ratios were identified as critical factors controlling methane activation and the methanol yield. Extension of the study to ethane (C2) showed moderate yields of methanol (3.6%) and ethanol (4.5%).

## Introduction

Indirect, high temperature (600–1100 °C) steam reforming coupled with high pressure (400–800 psi) syngas conversion using Cu/ZnO/Al_2_O_3_ catalysts afford methanol^1^. Direct partial oxidation at mild temperatures (< 450 °C) are afforded as two main strategies used in conversion of methane to methanol^2–5^. Oxidation of methane to methanol using O_2_ and H_2_ is known to produce methanol in water^6–10^. Even though the direct partial oxidation of methane is thermodynamically feasible, the overoxidation of subsequent products such as methanol, formaldehyde and formic acid to CO_2_ have less activation barrier than activation of methane^11–13^.

In the direct route, a trade-off between methane bond activation (E_a_ 175 kJ/mol on Cu(111)) and product (methanol and other oxygenates) protection (methanol has ~ 50 kJ/mol lower bond C–H dissociation energy than methane) against overoxidation govern the overall yield of methanol^3,11,14^. Based on the above concept, Nørskov et al. recently established a mathematical model^11^ to explain the reason for low methanol yields (< 1%) despite years of research. This model recognizes solvation free energy (SFE) modification of methanol as one approach to improve product yield by decreasing the activation free energy difference between methane and methanol (product protection). Similarly, minimization of mass transfer limitations (MTL) in conventional homogenous catalytic systems which arise due to limited solubility of oxygen and methane, may lead to improved methanol yields (methane activation). These low solubility and mass transfer limitations can be avoided by going into a supercritical or near critical phase. Unusual selectivities were previously observed by Debendetti et al.^15^ for toluene disproportionation over ZSM-5 that was ascribed to near critical clustering. The authors hypothesized the near critical clustering of toluene resulted in more surface reactions as the diffusion inside the zeolite pores was reduced. For two phase reaction systems, the near critical clustering phenomenon is not hitherto explored. We have studied a number of two phase systems comprising of solvents such as CO_2_^16^ and acetonitrile. We observed acetonitrile-O_2_ based system showed unusual changes in reaction selectivity for partial oxidation of methane above the critical point of acetonitrile.

Savage et al. studied methane to methanol and methanol overoxidation reactions in near and supercritical (NSC and SC) water^17–19^. Water at critical conditions behaves similarly to a nonpolar solvent with higher methane and oxygen solubility which could relax the aforementioned MTL during the reaction^20^. If insignificant MTL are assumed, the bulk could be enriched with methane and oxygen which sets conditions for the overoxidation reaction. We have explored a number of super critical and near critical solvent systems (Supplementary Table S1 for scCO_2_, FCH_2_CN, Cl_3_CCN, water, benzene). We observed unusual selectivity of methanol formation at short residence times with weakly hydrogen bonding nitrile solvents. Weak hydrogen bonding aprotic solvents such as acetonitrile can form molecular clusters at critical conditions which can act as localized reaction pockets isolated from the bulk^21,22^.

Even though SC solvents (e.g. water) can activate methane^23,24^, introduction of secondary activators may be required to improve the product yield^25^. Oxidative dehydrogenation, selective oxidation, and the coupling ability of boron based catalysts such as borocarbonnitrides^26–28^ and hexagonal boron nitride (*h*-BN)^29–31^ have been previously utilized in C1–C4 hydrocarbon to olefin conversion reactions. Although the C–H bond, molecular oxygen activation ability, and low carbon dioxide selectivity of B_2_O_3_ has been studied^32^, using *h*-BN have not been exploited in methane to methanol conversion reactions under SC conditions. In summary, SC solvent could minimize MTL (solubility), control local methane/oxygen concentrations (clustering), activate methane/oxygen, and protect methanol against overoxidation (clustering, SFE) while methanol formed on *h*-BN can be protected by SFE modification and controlled local oxygen concentration inside the cluster. Synergetic effects between modulation of local molecular concentrations by SC (275 °C, ~ 4000 psi) acetonitrile clusters to avoid the overoxidation reaction and methane activation by *h*-BN have been investigated in this study.

## Experimental

### Materials

All experiments were carried out in a 50 mL high-pressure (max 5000 psi) reactor model A3240HC6EB (Parr Instruments, Moline, IL) utilized with reactor controller 4848. The reactor controller was operated in the PID controlling mode and the rotor was at the highest rotation speed of 60 rpm. The system pressure and temperature were continuously digitally monitored with the associated software. All reactant gases including ultra-purity nitrogen, helium, oxygen, methane, ethane, carbon dioxide, and propane were purchased from Airgas, Inc, North Franklin, CT. Graphite, h-BN, acetonitrile, fluoroacetonitrile, trichloroacetonitrile, deuterated acetonitrile, and copper perchlorate hexahydrate were purchased from Sigma Aldrich. Liquid reactants were introduced with a 1000 μL micropipette (one-time use tips) and sampling was carried out with the help of single-use sterilized syringes and PTFE microfilters.

### Method

**Figure 1:**
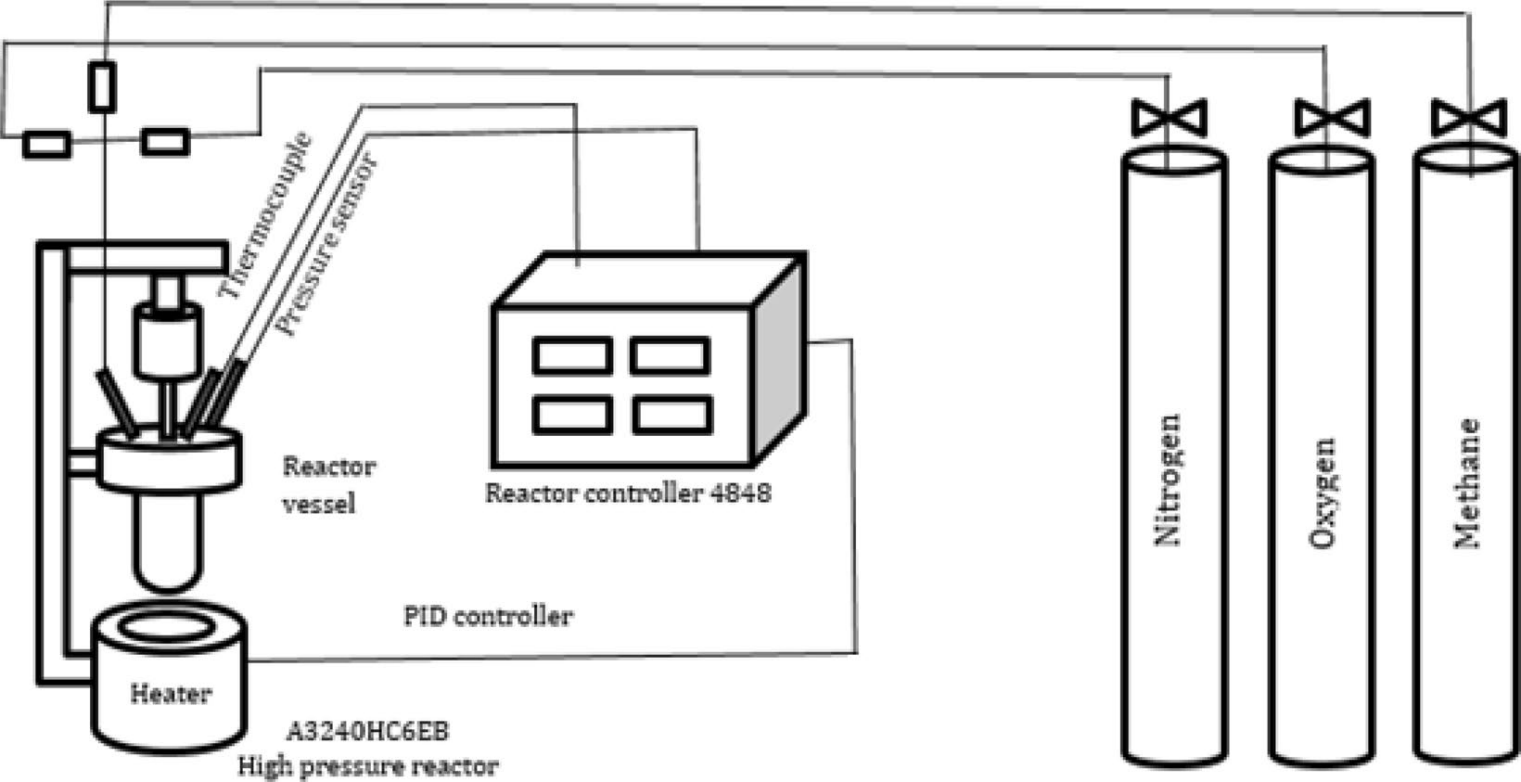
High-pressure reactor and setup design equipped with Parr 4848 reactor controller.

In a typical reaction, solvent and catalyst were loaded to the reactor. The reactor was cooled down to − 30 °C with liquid nitrogen and pressurized with the calculated amounts of O_2_, CH_4_, and inert gas (N_2_ or He) while the temperature was stable at − 30 °C. Then the reactor was heated to the precalculated (to avoid explosion range) temperature with a ramp rate of 2.5 °C/min (PID) and maintained there for the desired dwelling time. The reactor was then cooled down to ambient temperature by natural convection. Products were analyzed by GC–MS and NMR to determine the methanol concentration. Product mixtures were extracted with a single-use sterilized syringe and filtered with 0.22 μm PTFE microfilters. The complete reactor flow diagram can be found in Fig. 1.

Methanol and CO_2_ yield, and selectivity were calculated using following formulas (assuming CO_2_ is the only by product formed),

$$\text{Methanol yield CH}_{4}\text{ based }= \frac{moles\, of \,methanol}{moles\, of\, methane}\times 100\%,$$

$$\text{Methanol yield O}_{2}\text{ based }= \frac{moles\, of\, methanol}{moles\, of\, oxygen\times 2}\times 100\%,$$

$$\text{Methanol \,selectivity }= \frac{moles \,of\, methanol}{moles \,of\, methane\, reacted}\times 100\%,$$

$${\text{CO}}_{2}\,\text{ selectivity }=\frac{moles\, of \,\text{CO}2}{moles \,of\, methane\, reacted}\times 100\%,$$

$${\text{O}}_{2}\,\text{ conversion }=\frac{(moles \,of \,methanol+moles \,of\, \text{CO}2\times 2)}{moles\, of \,oxygen\times 2}\times 100\%.$$

## Results

### Optimization of the reaction parameters

#### Optimization of methane:oxygen molar ratio

**Figure 2:**
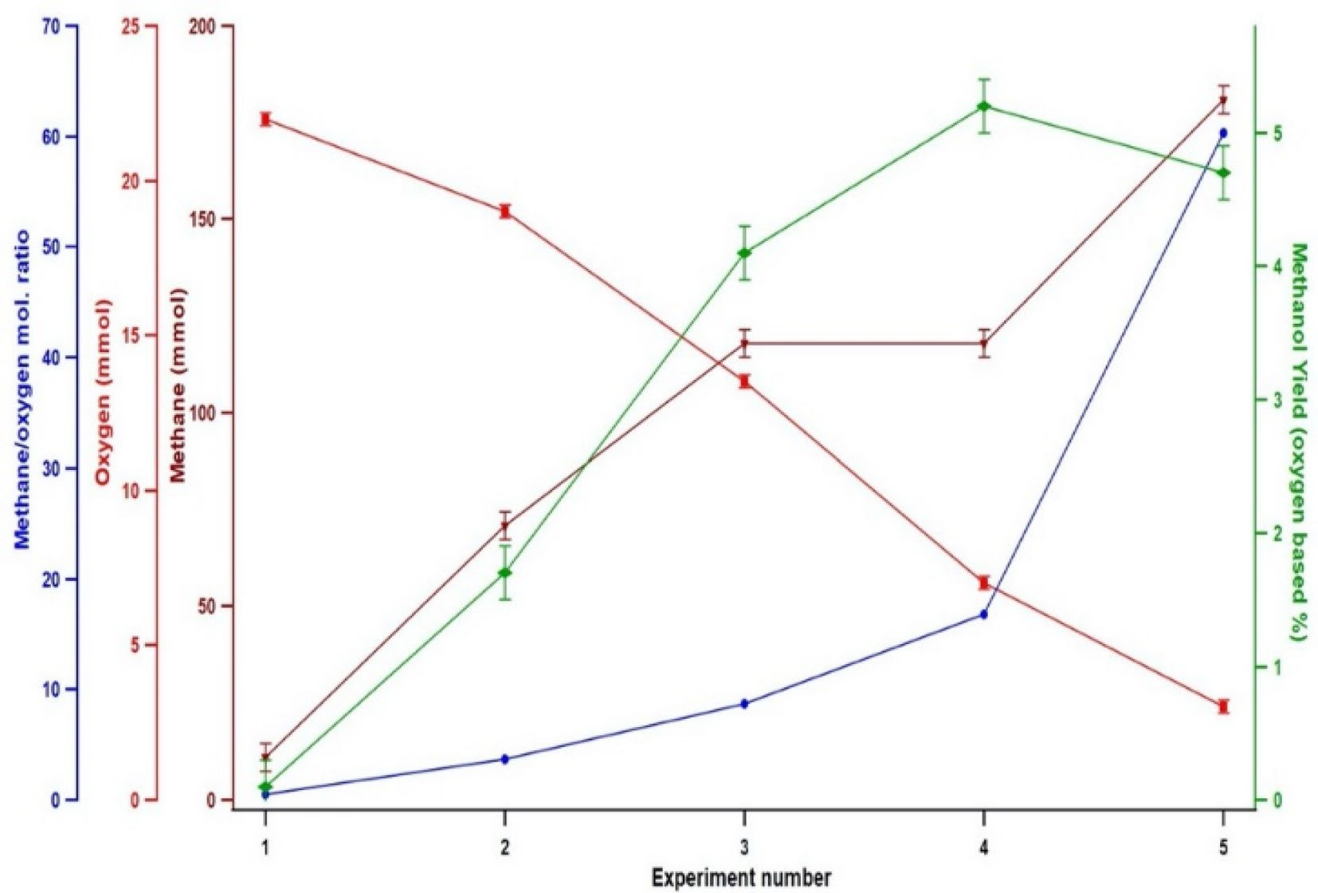
The effect of methane to oxygen ratio on direct methane oxidation to methanol in near supercritical acetonitrile. All entries were conducted with 3 mL (57 mmol) of acetonitrile loaded into the reactor and cold fed (− 30 °C) with desired molar amounts of O_2_, CH_4,_ and He. The reactor was heated with a ramp rate of ~ 2.5 °C/min up to 275 °C and the heater was programmed to switch off at 275 °C. A 60 rpm stirring speed was used. The samples were cooled to ambient temperature at the natural convection rate. Standard deviation (S. D.) was based on deviation in loading pressure. Methanol yield was calculated based on gas chromatography (GC) peak area calibration plots and reconfirmed with nuclear magnetic resonance (NMR) studies. The standard deviation of the yield was calculated by replicating one experiment and comparison with NMR data.

The methane to oxygen molar ratio was systematically increased by increasing methane and decreasing oxygen amounts to validate the product overoxidation hypothesis as shown in Fig. 2. An increase in the oxygen-based methanol yield from 0.1 to 5.2% was noticed as the methane/oxygen molar ratio was increased from 0.5 to 16.8. Even at lower oxygen (3 mmol) amounts, higher methanol yield (4.7%) was noticed. A significant increase (27%) in the methanol yield was noticed when the methane amount was maintained at a constant (118 mmol) value and the oxygen amount was decreased from 13.5 to 7 mmol, see Fig. 2 experiments 3 and 4. The increase in the yield was linear up to the molar ratio of 8.7 and then the yield started to fall at higher ratios. When the ratio was at 60, a decrease in the methanol yield to 4.7% was observed.

The headspace gas analysis of the reactor after the reaction showed only CO, CO_2_ permanent gases as shown in Figs. S5 and S6.

#### Optimization of the temperature

The effect of temperature on methanol yield was studied by maintaining an optimum methane/oxygen ratio of 16.8 and without any dwell time as shown in Table 1. An increase in the oxygen-based methanol yield from 0.3 to 6.6% was noticed when the reaction temperature was increased from 250 to 300 °C. The lowest carbon dioxide selectivity was observed when the reaction was performed at 275 °C. A 25 °C higher or lower reaction temperature than 275 °C resulted in higher carbon dioxide selectivity. The optimal reaction temperature was selected as 275 °C for the rest of the study.

**Table 1:** The effect of temperature on direct methane oxidation to methanol in near supercritical acetonitrile.

Cut off T (°C)	MeOH yield (%) CH_4_ based S.D. 0.4 × 10^–1^	MeOH yield (%) O_2_ based S. D. 0.2	CO_2_ yield (%) O_2_ based	MeOH Selectivity %	CO_2_ Selectivity %	O_2_ Conversion%
250	0.4 × 10^–1^	0.3	0.6	50	50	0.9
275	0.5	4.6	1.6	85	15	6.2
300^†^	0.8	6.6	53	20	80	59
275*	2.0	17	4.6	88	12	22

*With 400 mg *h*-BN (2.30: 1 h-BN: oxygen molar ratio), all other runs were conducted with 3 mL acetonitrile, 7 mmol of O_2_, 118 mmol of CH_4_, and 163 mmol He. All gases were cold fed to a total of ~ 1570 psi at − 30 °C. The reactor was heated at a 2.5 °C/min ramp rate to 275 °C. The heater cut off was set at 275 °C (no dwell time). The stirring speed was set at 60 rpm. The reactor was cooled to ambient at the natural convection rate. Standard deviations (S. D.) were based on deviations in loading pressure. Methanol yield was calculated based on a GC peak area calibration plot and reconfirmed with NMR. The standard deviation of the yield was calculated by replicating one experiment and comparing those data to NMR data. Small amounts of acetamide and acetic acid were formed as hydration products of acetonitrile and for simplicity purposes those products were excluded from the calculations.

^†^Higher tendency towards formation of acetamide and acetic acid relative to other reaction conditions was observed. As based on the GC traces these products were formed in negligible amounts for entries 1, 2 and 4.

#### Effects of different supercritical solvents and optimization of acetonitrile:oxygen molar ratio

Effects of different solvents on methane activation were studied as shown in Table S1. The molar ratio and solvent volume have been adjusted to avoid the flammability range of methane. Even though a direct comparison cannot be attained due to changes in conditions, a general idea about the reactivity trend can be obtained from these experiments. The methanol yield was halved (2.7%) when deuterated acetonitrile was used instead of non-deuterated acetonitrile (5.3%). Low methanol yield (1%) compared to deuterated or non-deuterated acetonitrile was noticed when a trichloroacetonitrile rich acetonitrile mixture was used in the reaction. Low methanol yields, 5% and 0.4%, were noticed when reactions were performed with conventional supercritical solvents such as carbon dioxide and water. The lowest methanol yield (0.04%) was observed in apolar solvent benzene as compared to other solvents.

**Figure 3:**
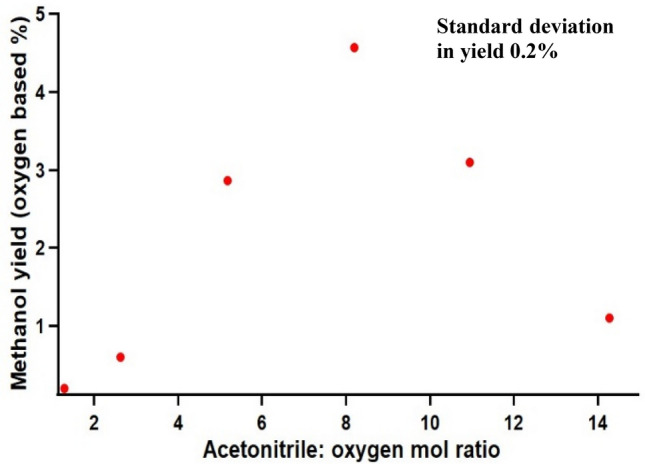
The effect of acetonitrile to oxygen molar ratio on direct methane oxidation to methanol in near supercritical acetonitrile. 9.5–95.7 mmol acetonitrile, − 30 °C cold feed, 163 mmol He, 120–124 mmol methane, 7.3–7.4 mmol of oxygen (without *h*-BN). All entries were carried out at 275 °C with 2.5 °C/min ramp rate, zero dwell time, and natural cooling to room temperature, 60 rpm stirring speed. Methanol yield was calculated based on the GC peak area calibration plot and reconfirmed with NMR. The standard deviation of the yield was calculated by replicating one experiment with comparison to NMR data.

The methane/oxygen molar ratio (same amount of methane and oxygen) was held constant at the optimum value (~ 17) while changing the acetonitrile content in the system at 275 °C as shown in Fig. 3. A linear increase in the methanol yield (O_2_ based) from 0.2 to 4.6% was noticed as the solvent to oxygen molar ratio was increased from 1.3 to 8.

#### Effects of different nitrides, stirring and optimization of *h*-BN:oxygen molar ratio

**Figure 4:**
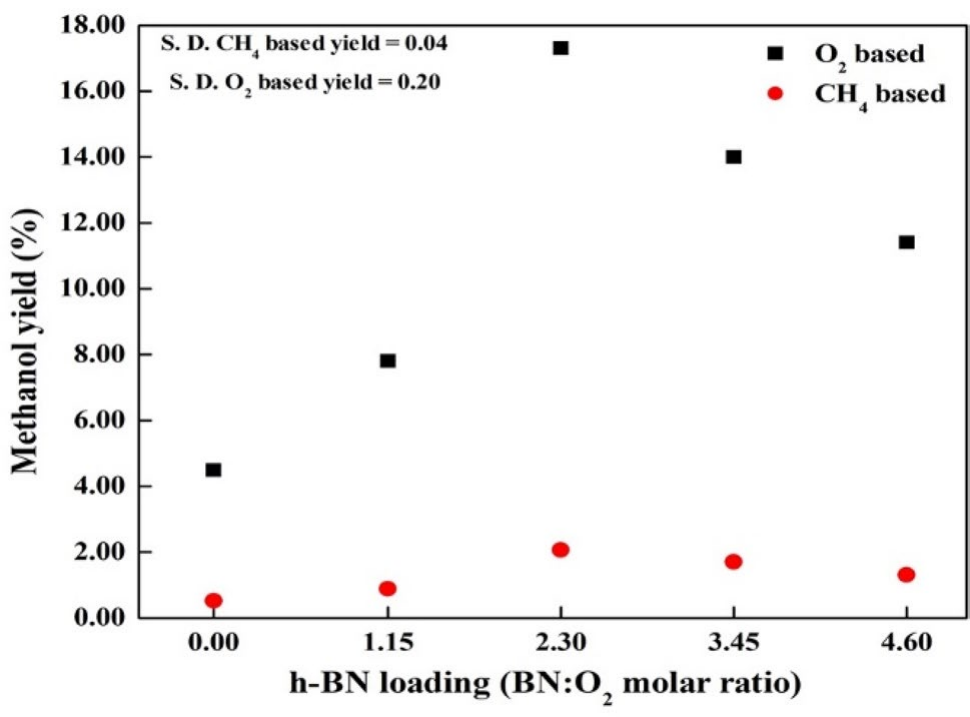
Variation of oxygen and methane-based methanol yield with different boron nitride loading. All runs were conducted with the specified amount of *h*-BN, 118 mmol of CH_4_, 7 mmol of O_2_, 163 mmol of He, 57 mmol acetonitrile heated up to 275 °C, no dwell time, 60 rpm stirring.

After optimizing the acetonitrile only conditions, *h*-BN was introduced into the system to investigate synergetic effects. A 70% (from 4.6 yield) increase in the methanol yield was observed when 1.15: 1 *h*-BN: oxygen molar ratio of hexagonal boron nitride was used as a catalyst (Table 1). Relatively low methanol yields were observed in the presence of other nitrides (Supplementary Table S2) including C_3_N_4_ (0.4%), InN (1.7%), and TiN (0.8%). A relationship between the system stirring and the methanol yield was observed as shown in Supplementary Table S3. The methanol yield was increased by 50% upon stirring the system at 60 rpm compared to the non-stirring system. A methanol yield of 3%, lower than the acetonitrile only yield (5.2%), was noticed even in the absence of acetonitrile and in the presence of *h*-BN. A 118% increase in the methanol yield was identified upon increasing the boron nitride loading to 2.30: 1 *h*-BN: oxygen molar ratio. A steady decrease in the methanol yield was observed when the boron nitride loading was increased from 2.30: 1 to 4.60: 1 (*h*-BN: oxygen molar ratio) as shown in Fig. 4. Both oxygen and methane-based methanol yields are depicted for comparison to the literature. A maximum oxygen-based methanol yield of 17.3% and methane-based yield of 2.0% were observed at 2.30: 1 *h*-BN to oxygen molar loading. Use of an oxygen-based yield can be justified by considering the limited oxygen (1: 17; oxygen: methane) amount in this study.

#### Optimization of acetonitrile:oxygen molar ratio with *h*-BN

**Figure 5:**
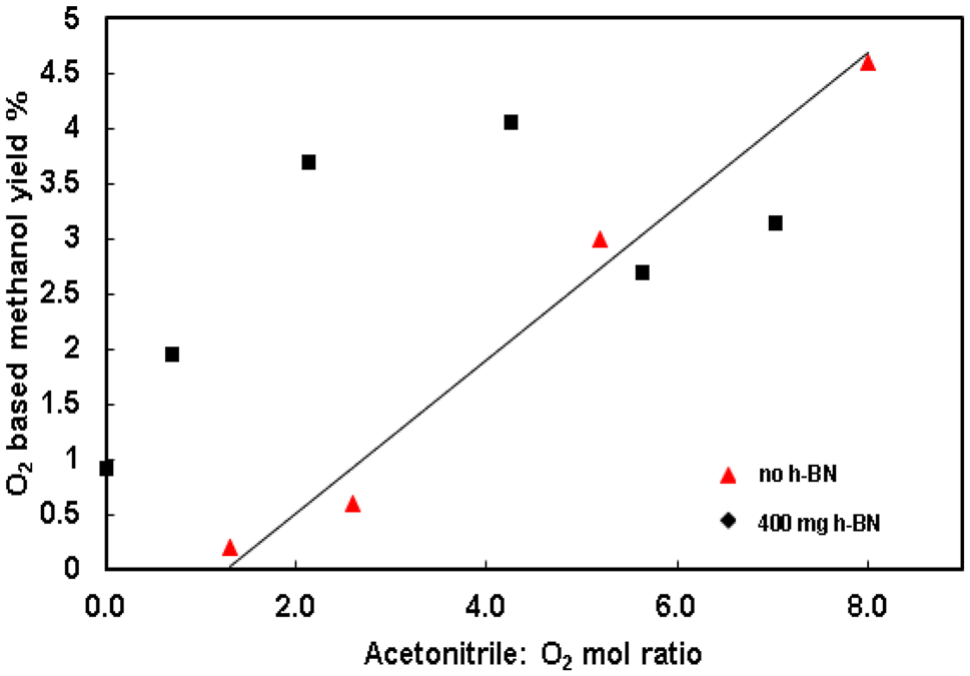
Variation of O_2_ based methanol yield with acetonitrile: O_2_ ratio and boron nitride loading. The graph does not illustrate 2.30: 1 *h*-BN to oxygen molar ratio, 8.2 MeCN: O_2_ ratio reaction which resulted in 17% yield. All runs were conducted with the specified amount of BN, cold feed; 118 mmol of CH_4_, 7 mmol of O_2_, 163 mmol of He. The amount of acetonitrile was varied (0 mmol, 19 mmol, 28 mmol, 57 mmol, 76 mmol, 95 mmol) and heated to 275 °C, with no dwell time, and 60 rpm stirring.

The acetonitrile to oxygen ratio was changed in the presence of *h*-BN initiator as shown in Fig. 5. A linear increase in the oxygen-based methanol yield with (up to the ratio of 2) or without *h*-BN was observed with an increase in the acetonitrile to oxygen ratio. At ratios higher than 2, a different trend was observed with 2.30: 1 *h*-BN: oxygen molar ratio. When the ratio is at 8.2 the highest methanol yield was observed with or without *h*-BN.

#### Effect of *h*-BN loaded on different supports

*h*-BN was loaded on different supports including Al_2_O_3_, graphite, SiO_2_, and TiO_2_ using a borane-amine adduct based incipient wetness impregnation method to check the effect on selectivity of the products formed and to increase the surface area of *h*-BN. Low methanol yields, 0.06 and 0.02% were observed when BN was loaded on γ-Al_2_O_3_ and TiO_2_ respectively. When BN was loaded on silica or graphite, higher methanol yields were observed (3.6% and 3.9% respectively) as shown in Table S4. There were no significant changes in the selectivity of the products with the change of the support.

#### Extension to C2 activation

The substrate scope of the reaction was investigated with higher hydrocarbons (C2) as shown in Table 2. Both methanol and ethanol were observed in the presence of ethane and the collective yield (8.1%) was more than the methanol yield (6.5%) in the methane reaction.

**Table 2:** The substrate scope of sub-supercritical acetonitrile and boron nitride catalyzed methane activation on C1–C2 hydrocarbon.

Entry	Substrate	Methanol yield%	Ethanol yield%
1	Methane	6.5	n/a
2	Ethane	3.6	4.5

Yields calculated based on oxygen. Conditions vary. 57 mmol MeCN, 0.60: 1 *h*-BN: oxygen molar ratio (8.01 mmol *h*-BN and 13.5 mmol O_2_), 118 mmol CH_4_ or 15.8 mmol C_2_H_6_, 163 mmol He, 275 °C, no dwell time. (*n/a* not applicable).

### Isotopic label experiment

In order to eliminate the assumption of methanol formation from solvent acetonitrile, ^13^C labeled methane was used for the experiment while keeping other parameters constant (400 mg *h*-BN, 3 mL acetonitrile, 7 mmol of O_2_, 118 mmol of ^13^CH_4_ (99% pure), and 163 mmol He. All gases were cold fed to a total of ~ 1570 psi at − 30 °C. The reactor was heated at a 2.5 °C/min ramp rate to 275 °C. The heater cut off was set at 275 °C (no dwell time). The stirring speed was set at 60 rpm). After cooling down the reactor the filtered solution was injected to GC–MS to analyze the formed products.

### Pre- and post-catalytic studies

**Figure 6:**
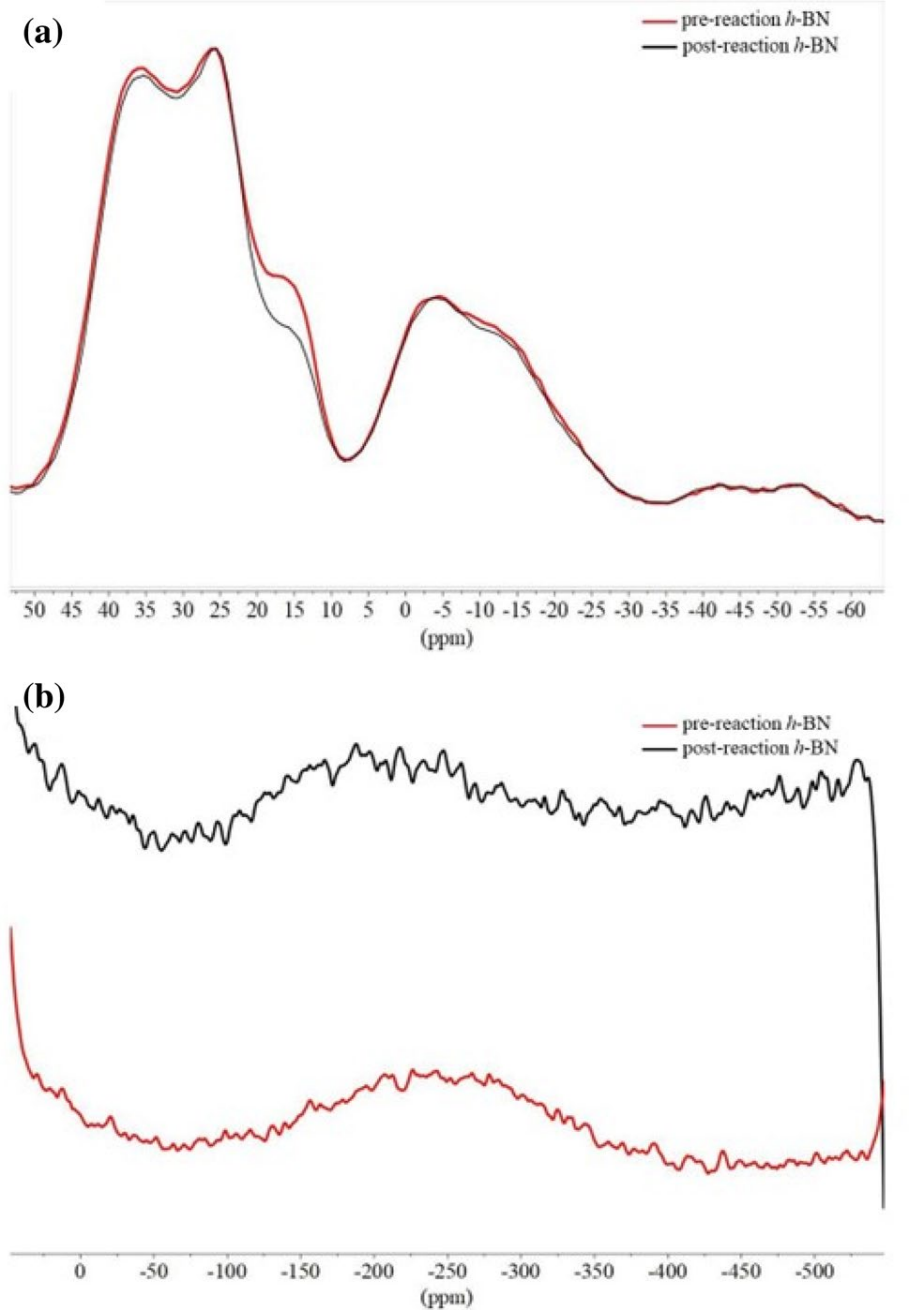
(**a**) MAS ^11^B SS-NMR spectra, (**b**) ^15^N SS-NMR spectra of pre- and post-reaction *h*-BN. Reaction conditions used; 400 mg *h*-BN, 118 mmol CH_4_, 7 mmol O_2_, 163 mmol He, 57 mmol acetonitrile, 275 °C, no dwell time, and 60 rpm stirring.

According to the Auger electron spectroscopic (AES) data a slight decrease in the intensity of the oxygen KLL signal in the post-reaction *h*-BN catalyst was seen in comparison to the pre-reaction *h*-BN (Fig. S3). ^11^B solid state NMR (SS-NMR) data also indicated the decrease in the B(OH)_x_O_3-x_ bond intensity at 15.5 ppm (Fig. 6a, Table 3) upon comparison between pre- and post-reaction *h*-BN^33^. Furthermore, post-reaction *h*-BN ^15^N SS-NMR showed a downshift in comparison to the pre-reaction *h*-BN (Fig. 6b).

**Table 3:** Summary of experimentally measured and literature ^11^B SS-NMR parameters.

Material	Assigned site	(ppm)	Reference
**This study**			
*h*-BN	BH_x_N_3−x_*	36.7	–
	BN_3_	26.1	–
	B(OH)_x_O_3−x_	19.0–11.0	–
**Literature**			
*h*-BN	BN_3_	30.4	^33^
	B(OH)_x_O_3−x_	19.2–11.5	
Borax	B(OH)_3_	19	^34^
B_2_O_3_	BO_3_	14.6	^35^
Poly(aminoborazine)	BHN_2_	31	^36^
	BN_3_	27	

*The assigned state was determined by closest literature reported value for BN system.

**Figure 7:**
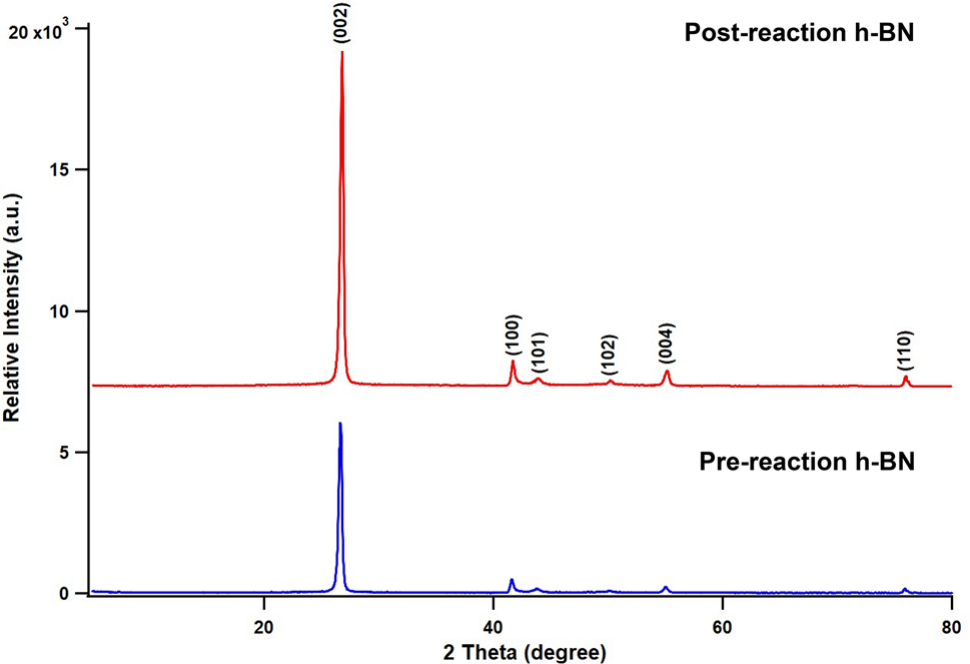
X-ray diffraction analysis of pre- and post-reaction h-BN. Reaction conditions used; 400 mg *h*-BN, 118 mmol CH_4_, 7 mmol O_2_, 163 mmol He, 57 mmol acetonitrile, 275 °C, no dwell time, and 60 rpm stirring.

The X-ray photoelectron spectroscopy (XPS) analysis showed an increase in O–H%, decrease in O–B%, increase in N–B%, increase in N–H%, increase in B–N%, and decrease in B–O% in post-reaction *h*-BN compared to pre-reaction *h*-BN^37–40^. The binding energies of both B and N of post-reaction *h*-BN shifted to lower binding energies compared to pre-reaction *h*-BN (Table S5). The crystallinity of the *h*-BN was increased after the reaction when compared to pre-reaction *h*-BN (Fig. 7).

## Discussion

Control of the available amount of oxygen is critical since oxygen is responsible for both methane conversion and product overoxidation^41^. Sato et al. used a lower O_2_/CH_4_ molar ratio (0.03) for methane conversion in supercritical water^42^. The methane to oxygen ratio study showed that relatively higher oxygen amounts (22 mmol) lead to lower methanol yields (Fig. 1) probably due to product overoxidation. As the oxygen amount decreased (22 mmol to 7 mmol) the available oxygen molecules that participate in both overoxidation and conversion reactions may have been diminished which would result in a tradeoff in yield. Increase in the yield even upon decreasing the oxygen content (constant methane) suggests that at lower oxygen partial pressures the methane activation reaction surpasses the overoxidation^3^. Another explanation can be introduced in terms of the supercritical solvent clustering effect explained elsewhere^21^. Methane can be converted to methanol inside the acetonitrile cluster by activated oxygen, and methanol can be subsequently released from the cluster into an oxygen-rich bulk solution where the oxidation takes place. As the oxygen content decreased the spectating oxygen in the bulk can be decreased which results in improved yield. At even lower oxygen content (3 mmol) oxygen could be acting as a limiting reactant inside the cluster resulting in slightly lower yield (4.7%) compared to 7 mmol reactions (5.2%). Even though evidence to isolate the exact mechanism has not been established, both these explanations stem from the overoxidation hypothesis.

Experiments conducted at three temperatures; below (250 °C), just above (275 °C), and above (300 °C) critical temperatures further corroborate the overoxidation phenomena. When the solvent temperature stays below the critical temperature, the clustering effect can be limited which would result in lower to no methanol yield (0.3%) due to overoxidation. Higher carbon dioxide selectivity at 250 °C (50%) compared to 275 °C eliminates the possibility of lack of enough energy for activation. Just above supercritical conditions, 275 °C, CO_2_ selectivity decreased to 15% suggesting product protection by cluster formation at supercritical conditions. Even though more yield through cluster formation is possible at 300 °C, higher temperatures may have contributed to the total combustion of methane to carbon dioxide (80% CO_2_ selectivity).

The static dielectric constant and density of acetonitrile depend on the temperature and pressure (reduced density) of the system^43,44^. As temperature increases, density and dielectric constant decrease whereas pressure has a completely opposite effect. At the critical point (272.35 °C, 703.43 psi) the density of acetonitrile was reported as 0.225 g/mL whereas the density of methane, methanol, benzene, carbon dioxide, and nitrogen are 0.162, 0.270, 0.210, 0.225, 0.313 g/mL respectively^44^. At critical pressure, the dielectric constant of acetonitrile is 40.3 (25 °C) and at critical temperature extrapolated values go down to 0.66 (at 1 bar)^45^. Due to the above reasons, acetonitrile is expected to behave as an apolar gaseous solvent with high apolar compound solubility (10^3^ to 10^8^) which can facilitate methane solubility^46^. Organic solvents in high-pressure or supercritical pressure can arrange into high density localized microreactor pockets due to high compressibility^47,48^. The size of these clusters can be altered based on the pressure of the system^48^. Oxygen and methane can be activated inside the apolar clusters via strong solvent-gas interactions and product molecules can be protected against overoxidation. We have probed high pressure differential scanning calorimetry (DSC) of acetonitrile and O_2_ under the same reaction conditions as for methane partial oxidation and have seen no oxidation of acetonitrile at short residence times. We have also probed the potential of clustering phenomena by using diffusivity measurements via high pressure NMR. These clustering phenomena seem to be dynamic and shorter than the NMR timescale. NMR chemical shift measurements show that the acetonitrile remains unchanged under the reaction conditions in the presence of O_2_.

Strong interactions between oxygen/methane and acetonitrile clusters are believed to activate the gaseous reactant molecules. As the solvent to oxygen ratio increased, the number of available solvent molecules to make strong interactions with solutes (methane, oxygen), hence clustering, would have been increased. Low product yield (0.2%) at low acetonitrile (10 mmol) concentrations could be attributed to insufficient cluster formation. The results infer that in order to form an adequate number of clusters, the solvent to oxygen ratio should exceed a critical value (in this case 5.2). The molar fraction of solvent (acetonitrile) in a mixture determines the critical temperature and pressure. As the acetonitrile molar fraction decreases, the critical temperature also decreases while the critical pressure can show a positively skewed curve^21^. If similar behavior is assumed, the critical condition (T, P) should be decreased compared to pure acetonitrile within the operating acetonitrile molar fraction regime of this study (0.03 to 0.17).

Use of acetonitrile as the subcritical solvent was justified by studying conventional supercritical systems such as water and carbon dioxide. The results showed that supercritical acetonitrile derivatives deliver higher methanol yields compared to conventional supercritical systems. All the reactions were performed at 275 °C (despite the critical point of water at 375 °C) due to significant methanol overoxidation at temperatures above 275 °C. The hydrogen-bonded cluster network of water decreases at supercritical temperature transforming into a tetrahedral packing structure^49^. The energetically most favorable five-membered cluster can be formed by hydrogen bonding between H and N of different acetonitrile molecules^50^. The hydrogen bonding strength, a common factor in both systems, can be disrupted by another hydrogen bonding additive/product. Electronegative -fluoro and -chloro derivatives of acetonitrile that have very similar boiling points (e.g., CH_3_CN 82 °C, FCH_2_CN 79–80 °C, Cl_3_CCN 83–84 °C) potentially impact the cluster properties and O_2_ solvation in a way that leads to lower methanol yields. Apolar carbon dioxide and benzene, on the other hand, can only participate in London dispersion forces and resulted in lower methanol yield (0.5% and 0.04% respectively). The critical points of benzene (289 °C) and acetonitrile (272 °C) are similar.

The 70% and 260% (from 4.6% yield, Fig. 3, Supplementary Table S3) increase in methanol yield (compared to no *h*-BN) with 1.15: 1 and 2.30: 1 *h*-BN: oxygen molar ratio of *h*-BN respectively, can be attributed to the hydrogen abstraction ability of *h*-BN and supercritical acetonitrile synergetic effects. Lower yield (0.4%) with carbon nitride (Table S2), a 2D structure similar to *h*-BN (Fig. S3), suggests a structure independent methane conversion pathway. Lower yields with other nitrides such as TiN, and InN, suggest unique chemistry of *h*-BN. The 50% increase (Table S3) in the methanol yield under stirring compared to no stirring typically suggests mass transfer limitations in the supercritical acetonitrile or *h*-BN system^29^. All reactions were performed at the maximum (60 rpm) stirring speed of the propeller to mitigate mass transfer limitations. This experiment demonstrated that even in the absence of acetonitrile, boron nitride itself could activate methane to methanol (3% yield) conversion under supercritical conditions. Addition of the yields only with BN (3%) and only with acetonitrile (4.6%) almost tally with the BN and acetonitrile yield (7.8%). The decrease in the methanol yield after 2.30: 1 *h*-BN: oxygen molar ratio of *h*-BN loading (Fig. 3) suggests mass transfer limitations in the system. The optimal solvent: oxygen molar ratio of 8.2 with or without *h*-BN further suggest a synergetic effect of supercritical acetonitrile and *h*-BN systems.

The doubt of methanol formation from the solvent acetonitrile rather than methane was cleared using ^13^C isotope labeled methane for the reaction while keeping other parameters unchanged. Upon analyzing the products using GC–MS, mass fragments for methanol peak m/z = 32 and 33 were shown as the highest abundant species respectively for ^13^CH_3_O^−^ and ^13^CH_3_OH fragments (Fig. S2).

**Figure 8:**
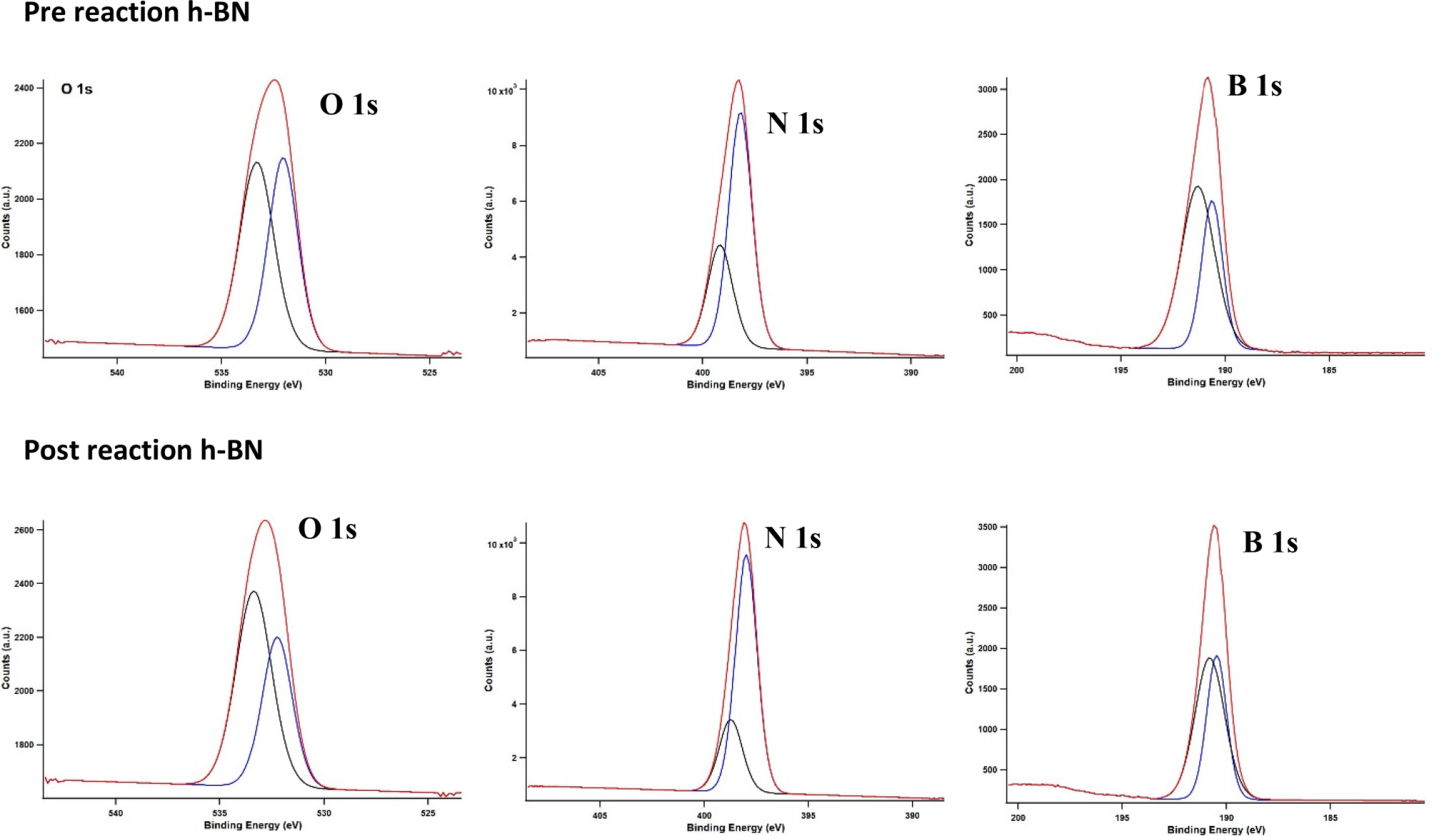
X-ray photoelectron spectroscopic (XPS) analysis of pre and post-reaction for *h*-BN obtained with Physical Electronics Quantum 200 scanning ESCA microprobe with Al Kα radiation (29.35 eV pass energy, charge compensated with adventitious carbon 284.8 eV).

The decrease in the intensity of the oxygen KLL peak of the post-reaction catalyst in comparison to the pre-reaction catalyst (Fig. S3) in the AES spectra could be due to oxygen terminated B–O bond dissociation during the methane oxidation reaction. Also, the decrease in the 15.7 ppm peak representing B–O in MAS ^11^B SS-NMR (Fig. 6a) upon comparison between pre- and post-reaction *h*-BN further confirm the observations shown from AES data. Furthermore, post-*h*-BN ^15^N-SSNMR showed a downshift in comparison to the pre-*h*-BN (Fig. 6b). This could be mainly due to the proton abstraction by N atoms in *h*-BN during the reaction. The observation of the decrease in the B–O (break in B–O–O–N bridge) after the reaction (Fig. 8) in XPS data agrees quite well with the AES and SS-NMR data. These data corresponded to a previously reported oxidative dehydrogenation (ODH) reaction mechanism (active site; an oxygen terminated armchair edge of BN bridge, B–O–O–N) of C2–C4 alkanes, to some extent^30^. However, methane conversion could not be explained with the ODH pathway. Methane to ethene oxidative coupling reaction mechanism on *h*-BN (active site; B–OH) suggested by Wang et al.^29^ could not explain the observations noted above, indicating that the active site for the reaction could be the terminal B–O–B bridge of h-BN. Based on the above facts, CH bond activation could occur on bridging oxygen sites to generate chemisorbed methoxy and hydroxyl groups (similar to the first step of ODH) which then desorbed to generate methanol. It is remarkable how the C2–C4 alkane ODH catalyst (*h*-BN)^51–53^ is also active for methane and ethane oxygenation reactions under supercritical conditions.

In most heterogeneous supercritical and subcritical thermal catalysis literature reports, methanol yield (methane-based) stays around 1%^23,54^ which showcases the uniqueness of the current study. The maximum carbon-based yield of 2% and oxygen-based yield of 17% under subcritical thermal catalysis have not been reported to date.

## Conclusions

In summary, a metal-free thermal catalytic partial oxidation of methane to methanol was developed using h-BN under near supercritical acetonitrile to lead to a yield of 17% (oxygen-based) of methanol. Furthermore, an extension of this method to ethane (C2) leads to 3.6% and 4.5% (oxygen-based) yields of methanol and ethanol respectively.

## Supplementary Information

Supplementary Information.

## Abbreviations

SFE: Solvation free energy
MTL: Mass transfer limitations
SC: Supercritical
NSC: Near supercritical
h-BN: Hexagonal boron nitride
GC: Gas chromatography
NMR: Nuclear magnetic resonance
DSC: Differential scanning calorimetry
ODH: Oxidative dehydrogenation
MAS: Magic angle spinning
