# Supplementary material for: Partial oxidation of methane to methanol on boron nitride at near critical acetonitrile
Source: Sci Rep. 2022 May 20;12:8577. doi: 10.1038/s41598-022-12639-x (PMC9122901; doi:10.1038/s41598-022-12639-x)
Supplement: Supplementary file 1 — Supplementary Information. [file 41598_2022_12639_MOESM1_ESM.docx]

Partial Oxidation of Methane to Methanol on Boron Nitride at Near Critical Acetonitrile

Tharindu Kankanam Kapuge^†^, Ehsan Moharreri^‡^, Inosh Perera^†^, Nicholas Eddy^‡^, David Kriz^†^, Nathaniel Nisly^†^, Seth Shuster^†^, Steven L. Suib^†‡*^, Partha Nandi^ǂ*^.

^†^Department of Chemistry, University of Connecticut, Storrs, CT 06269, USA. ^‡^Institute of Material Science, University of Connecticut, Storrs, CT 06269, USA.

^ǂ^Corporate Strategic Research, ExxonMobil Research and Engineering, Annandale NJ, 08801 USA.

**Supplementary Materials**

Methods


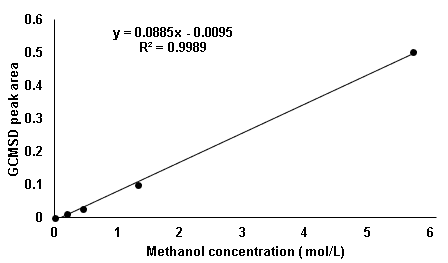
The headspace gas analyses were performed with the SRI GC equipped with TCD (150°C), two 12ʺ series HayeSep D columns, Ar carrier 20 sccm. The liquid samples were analyzed with the GCMSD equipped with a CP wax 52 CB column and NMR (400 MHz). Cyclohexanone was used as an internal standard in the GCMSD analysis and methanol calibration plot was constructed as shown in Supplemental figure 2.

**Figure S1.** GCMSD based methanol calibration plot constructed to determine the concentration of methanol in each reaction. The calibration plot was updated frequently to compensate for the changes in the GCMSD.

**Table S1.** The effect of solvent on direct methane oxidation to methanol in near supercritical conditions

| **Entry** | **Solvent** | **Final temperature (**°C**)** | **MeOH yield (%) O_2_ based** |
| --- | --- | --- | --- |
| 1 | MeCN | 300 | 5.2 |
| 2 | d3- Acetonitrile | 300 | 2.7 |
| 3 | Fluoroacetonitrile | 300 | 4 |
| 4 | Trichloroacetonitrile | 300 | 1 |
| 5 | Water | 300 | 0.4 |
| 6 | CO_2_ | 275 | 0.5 |
| 7 | Benzene | 275 | 0.04 |

Entry 1, 2; 57 mmol solvent, Entry 3; 17 mmol (0.95 mL) solvent. Entry 4; 7 mmol (0.3 mL) solvent and 51.7 mmol (2.7 mL) acetonitrile, total of 3mL solution. All above cold fed at -30°C, 7 mmol of O_2_, 118 mmol CH_4_, 163 mmol inert gas, 2.5°C/min of ramp rate up to 300°C, no dwell time. Entry 5; 3 mL of water (166 mmol), cold feed -30°C, 22 mmol of O_2_, 72 mmol of CH_4_, and 114 mmol of N_2_, 2.5 °C ramp rate up to 300 °C, 3 h dwell time. Entry 6; cold feed -30°C, 57 mmol of CO_2_, 118 mmol of CH_4_, 7 mmol of O_2_, 103 mmol of He, 2.5°C ramp rate heated up to 275°C. Entry 7; 3 mL benzene (33.6 mmol), -30°C cold feed, 7 mmol O_2_, 118 mmol CH_4_, 150 mmol He, 2.5 °C ramp rate heated up to 275 °C. All reactions were at cold fed final pressure 2100 psi and 60 rpm stirring speed.

**Table S2.** Use of different nitride initiators with super-critical acetonitrile

| Entry | Initiator | MeOH yield (O_2_ based) |
| --- | --- | --- |
| 12 | Carbon nitride | 0.4 |
| 13 | Indium nitride | 1.7 |
| 14 | Boron nitride | 7.8 |
| 15 | Titanium nitride | 0.8 |

200 mg initiator, 57 mmol acetonitrile loading. Cold feed (-30°C) 7 mmol of oxygen, 118 mmol methane, 163-197 mmol of He, Reactions were carried out at 275°C with 2.5°C/min ramp rate, no dwell time and 60 rpm stirring.

**Table S3.** The effect of boron nitride amounts as an initiator, acetonitrile amounts and stirring rate for methane to methanol conversion near supercritical conditions

| **Entry** | **BN amount (mg)** | **MeCN (mmol)** | **Stirring (rpm)** | **MeOH yield (%)**  **O_2_ based** |
| --- | --- | --- | --- | --- |
| 175 | 200 | 57 | 0 | 5.2 |
| 176 | 200 | 57 | 60 | 7.8 |
| 177 | 200 | 0 | 60 | 3 |
| 186 | 400 | 57 | 60 | 17 |

All runs were conducted with the specified amount of BN, 118 mmol of CH_4_, 7 mmol of O_2_, 163 mmol of He, 57 mmol acetonitrile heated up to 275°C, no dwell time, and 60 rpm stirring.

**Table S4.** Effect of different supports of *h*-BN on the methane to methanol conversion near supercritical conditions

| **Entry** | **Initiator** | **MeOH yield (%) O_2_ based** |
| --- | --- | --- |
| 213 | BN@γ-Al_2_O_3_ | 0.06 |
| 217 | BN@-SiO_2_ | 3.6 |
| 218 | BN@-TiO_2_ | 0.02 |
| 221 | BN@graphite | 3.9 |

200 mg initiator, 57 mmol acetonitrile loading. Cold feed (-30°C) 7.8 - 13.5 mmol of oxygen, 117-128 mmol methane, 163-197 mmol of He, Reactions were carried out at 275°C with a 2.5°C/min ramp rate.


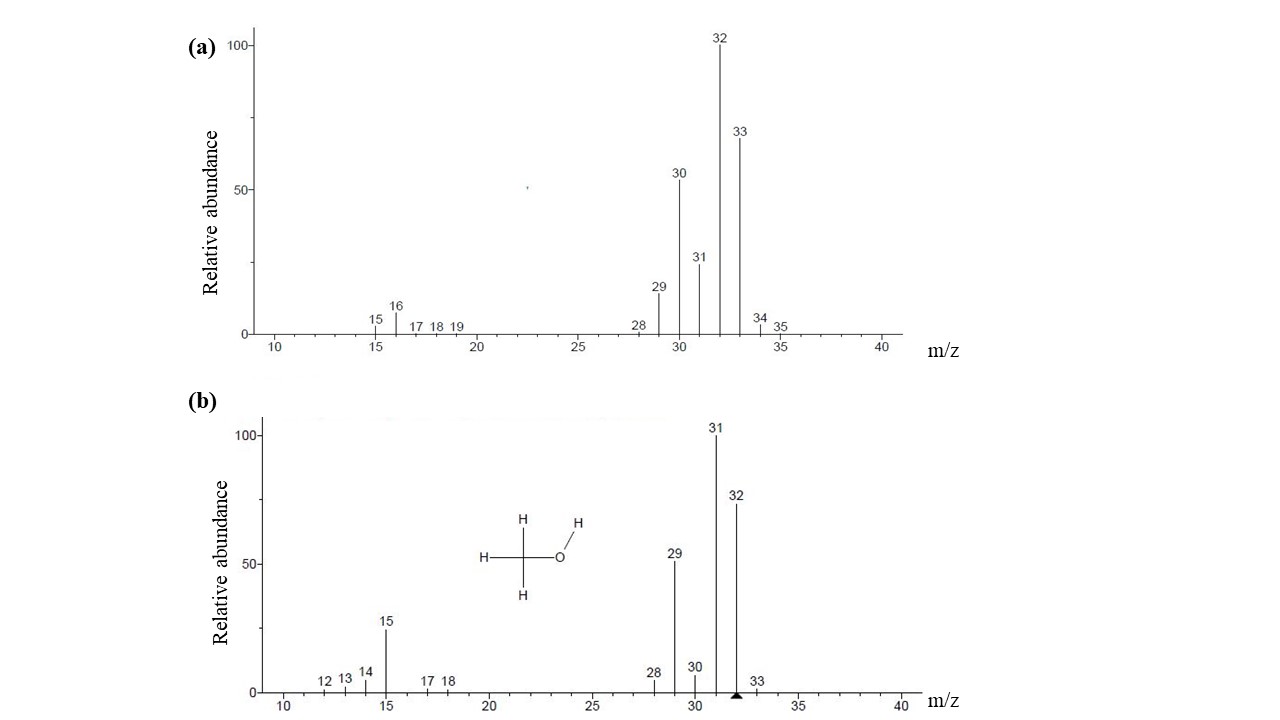


**Figure S2.** Mass spectra for ^13^CH_4_ isotopic label experiment (a) Mass fragements for the methanol peak (b) Mass fragments for methanol in natural occurance


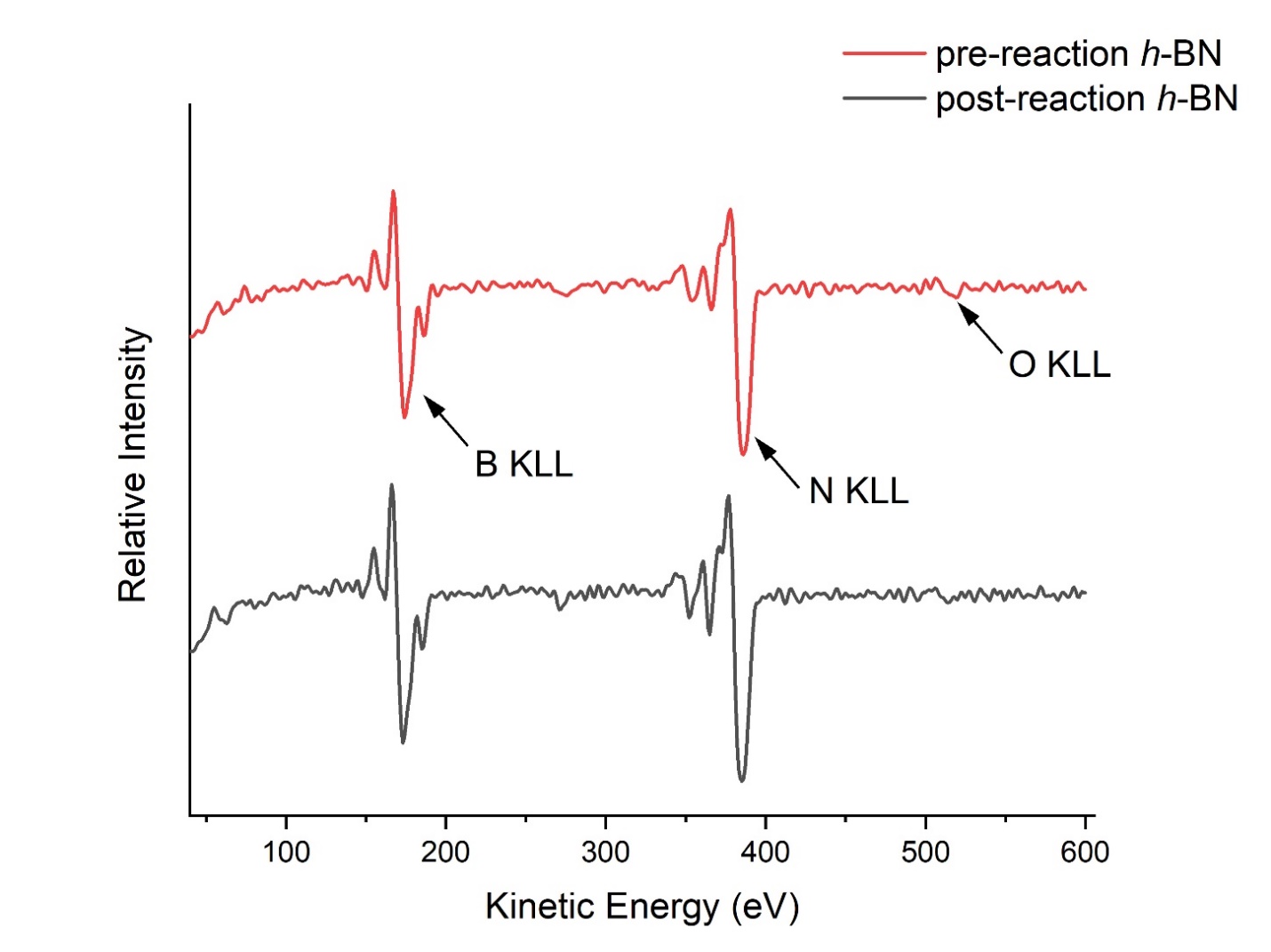


**Figure S3.** AES analysis of pre- and post-reaction h-BN. Reaction conditions used; 400 mg h-BN, 118 mmol CH_4_, 7 mmol O_2_, 163 mmol He, 57 mmol acetonitrile, 275°C, no dwell time, and 60 rpm stirring.

**Table S5.** The XPS peak positions and peak areas of pre and post-reaction h-BN

| **Sample** | **O position eV** | **O peak area** | **N position eV** | **N peak area** | **B position eV** | **B peak area** |
| --- | --- | --- | --- | --- | --- | --- |
| **Pre**  **h-BN** | 532.25  (OH)  533.37  (OB) | 53.65  (39%)  82.92  (61%) | 398.19  (NB)  399.16  (NH) | 641.63  (68%)  302.98  (32%) | 190.61  (BN)  191.29  (BO) | 118.46  (35%)  218.98  (65%) |
| **Post h-BN** | 532.05  (OH)  533.30  (OB) | 64.65  (47%)  73.19  (53%) | 397.98  (NB)  398.72  (NH) | 631.37  (75%)  206.57  (25%) | 190.46  (BN)  190.79  (BO) | 122.71  (40%)  180.40  (60%) |

The XPS data analysis showed an increase in O-H %, decrease in O-B%, increase in N-B %, increase in N-H%, increase in B-N%, and decrease in B-O% in post-reaction h-BN compared to pre-reaction h-BN. The binding energy of both B and N of post-reaction h-BN shifted to lower binding energy compared to pre-reaction h-BN.


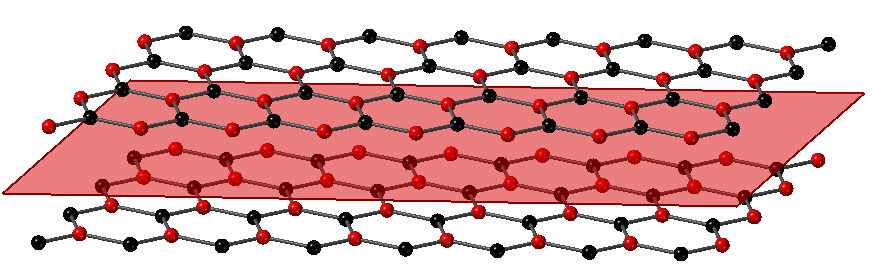


**Figure S4.** The structure of hexagonal BN showing the plane corresponding to the highest intense XRD peak (002)

Time (min)

Abundance


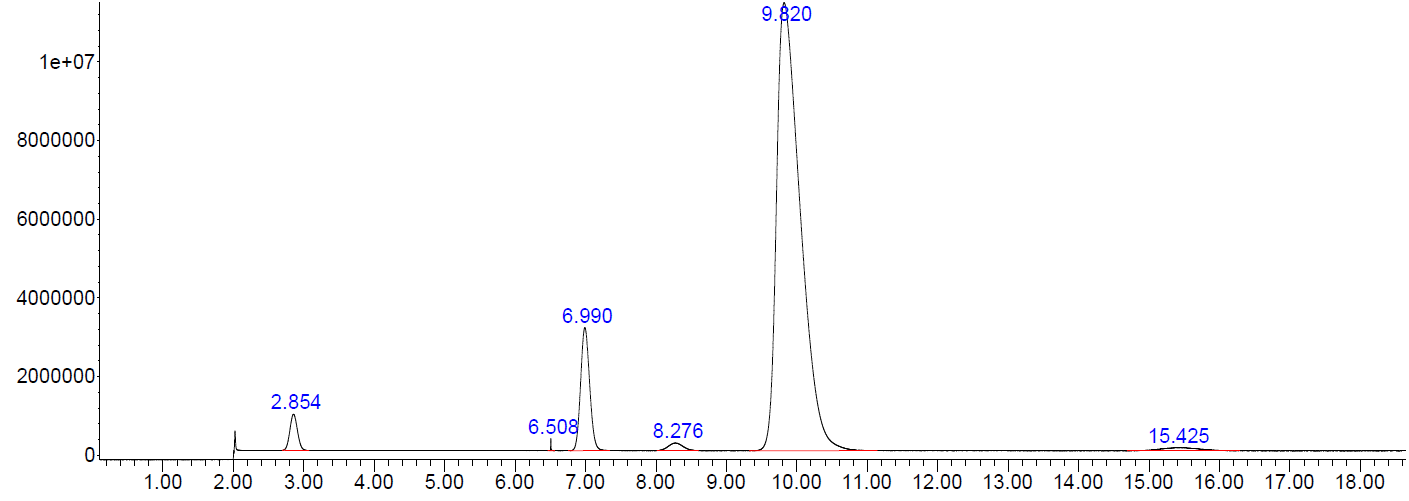


Abundance


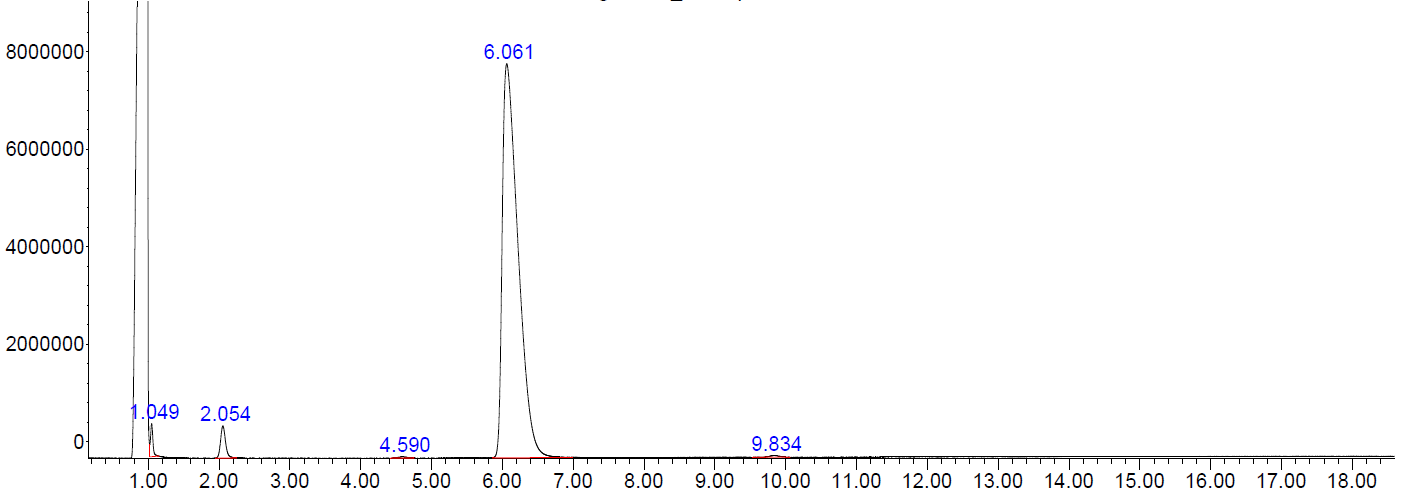


**TCD Helium channel**

CH_4_

Ar/O_2_

CO_2_

N_2_

CO

CO

CH_4_

N_2_

O_2_

H_2_

**TCD Argon channel**

Time (min)

**Figure S5.** Headspace GC analysis of the optimized high-pressure reaction (400 mg h-BN, 118 mmol CH_4_, 7 mmol O_2_, 163 mmol He, 57 mmol acetonitrile, 275°C, no dwell time, and 60 rpm stirring) with GC.

**Figure S6.** Headspace residual gas analysis (RGA) of the optimized high-pressure reaction (400 mg h-BN, 118 mmol CH_4_, 7 mmol O_2_, 163 mmol He, 57 mmol acetonitrile, 275°C, no dwell time, 60 rpm stirring) with a residual gas analyzer to eliminate NOx formation hypothesis from h-BN during the reaction. The reactor was cool down to -30°C in order to avoid liquid product flow.

**C**

**CH**

**CH_2_**

**CH_3_**

**CH_4_**

**OH**

**H_2_O**

**H_2_**

**H**

**He**

**N_2_/CO**

**O_2_**

**CO_2_**

**Ar**

**Ar**

**O_2_**

**N_2_**

**N**

**O**

**H_2_O**


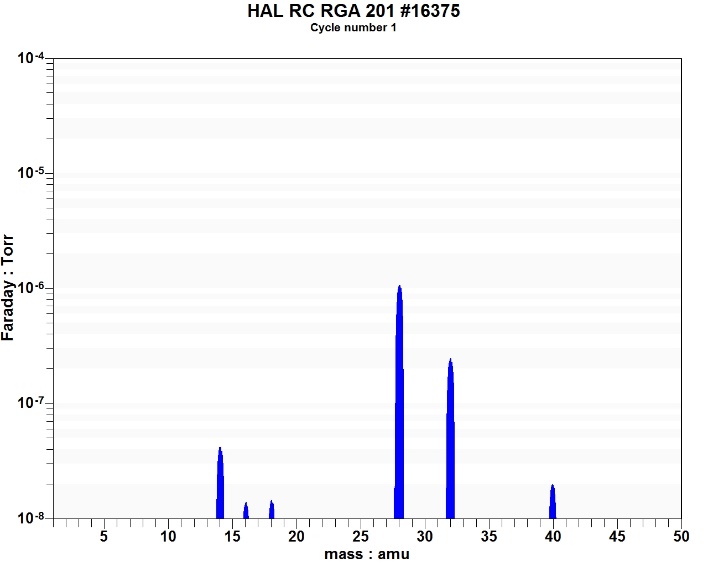

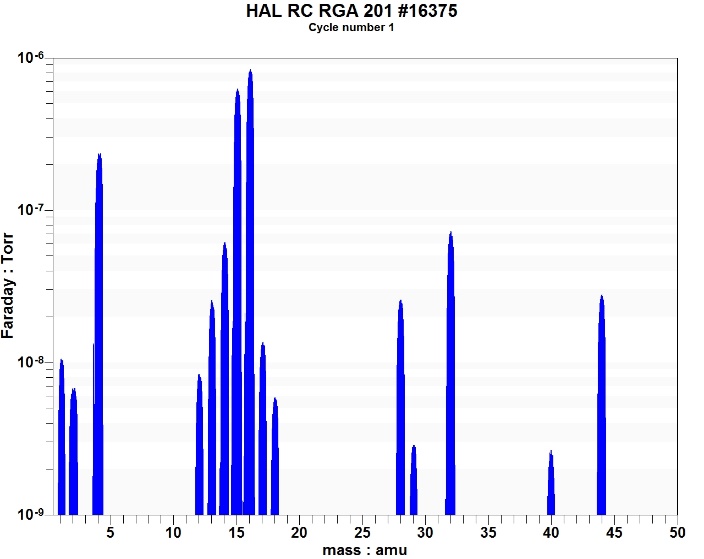


**Blank scan**

**Head space analysis**

**
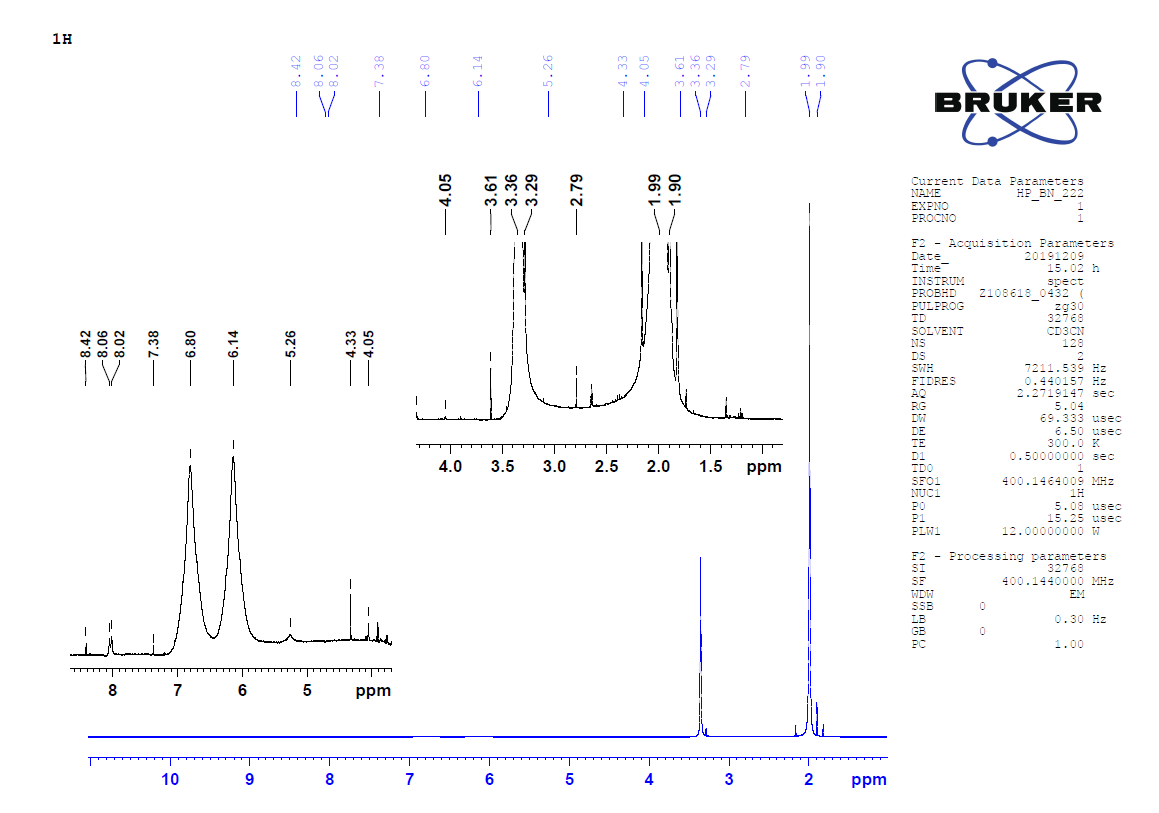
**

**CH_3_CN**

**CH_3_OH**

**Figure S7.** ^1^H NMR analysis (CD_3_CN, 400 MHz) of product mixture of supercritical methane to methanol conversion reaction. 200 mg *h*-BN, CH_4_, 7 mmol O_2_, 163 mmol He, 57 mmol acetonitrile, cold feed -30°C, 275°C, no dwell time, 60 rpm stirring.

Methanol hydroxyl and water protons have been exchanged (deuterium) with CD_3_CN. Therefore, peaks corresponding to H_2_O at 4.8 ppm and CH_3_OH at 4.26 were not observed in the NMR.
